# Mr. Charles Bell on Injuries of the Spine and of the Thighbone

**Published:** 1825-07-01

**Authors:** 


*Observations on Injuries of the Spine and of the Thigh Bone: in Two Lectures, delivered in the School of Great Windmill Street. The first in vindication of the Author's Opinions against the Remarks of Sir Astley Cooper, Bart. the second, on the late Mr. John Bell's Title to certain Doctrines now advanced by the same Gentleman.* By Charles Bell, Surgeon to the Middlesex Hospital. One Vol. 4to. pp. 101, Nine Plates, price 16s. bds. London, 1824.

"These two Lectures," says Mr. Bell, "are in some measure controversial; a manner of treating subjects of science, which,

---

* Dr. Latham's happy mode of exposing the absurdity of the Extra-professional Censor's Strictures, will be seen in the following passage.

"But let it be admitted, not only that flux of the bowels was the feigned disease of the Penitentiary, during six years and a half, but also, that feigned as it was, Mr. Pratt went on prescribing chalk mixture and chalk powder for it, as if it had been real during the whole of this period: what follows? A coincidence of the most extraordinary kind, namely, that this same disease which had been the *unreal* and *fictitious* disease of the Penitentiary during so many years, became all at once so unquestionably *real,* that the lives of half the prisoners were in jeopardy from it, and many actually died; and, moreover, that the very remedy which had been prescribed during so many years, *for no purpose whatever,* was the same which, at length, was found *most necessary and indispensable.*" 252.

whether interesting or not to the profession, is never agreeable to the public, who cannot comprehend, and have no sympathy with professional feelings." We are rather at a loss to understand the bearing of this sentence. What does Mr. Bell mean by the *public?* Surely he must mean the *professional public;* for it is not likely that any other will read a book on injuries of bones. The sentence, however, contains a most important truth —namely, that the public (meaning always the professional public) are rarely interested in the controversies of individuals, especially controversies respecting personal claims or priority of discoveries. We do not blame individuals, however, for asserting their rights, or supposed rights. All we mean to say is, that the authors of these controversies must lay their accounts with exciting but little interest (comparatively with their own feelings) in the minds of their readers. Under this impression, we shall, as usual, dwell principally on points of practical knowledge in this volume, and meddle little with litigation. Sir Astley Cooper and Mr. Bell are—

" ——————— Arcades ambo,
" Et cantare pares, et respondere parati."

Mr. Bell has offered some judicious reflexions in his preface. He very properly advises students not to pin their faith upon any individual master, school, or hospital, as is too often the case; but to look around them, and particularly to learn what our predecessors have said and done.

" The neglect of the literature of the profession deprives the student of all enthusiasm and love for it; he is brought up deficient in liberal views, and is taught to over-rate the importance of the person under whom he is educated, and to content himself with walking in the trammels of his particular practice.

" We hear a great deal of the improvement of surgery in modern times; but it appears to me that we lose, of what is old and good, fully as much, as we gain by our search after novelty; and those who are ignorant of the history of the art, and not aware of the observations and discoveries of the great men who have preceded them, are in continual danger, in straining after new inventions, of only restoring what has been discovered, tried, and rejected before their time." v.

Mr. Bell justly observes, that Mr. Hunter has done harm as well as good to surgery. That gentleman having been defective in his own education, "had the hardihood to assert that reading was folly." " So it has happened, that whilst the philanthropist and philosopher find an example in him, the vulgar and the ignorant think they discern their own characters in his." We hope and believe that a new era, in this respect, is at hand. The general spread of knowledge and the rage for reading, are now so extensive among all ranks, that *self* will induce the student to read and study, in order to keep his level in society. This is no longer, in fact, an optional matter with the rising generation—it is a matter of imperious necessity.

1. *Lecture on the Spine.—Fracture.* When called to a man with fractured spine, we are to inquire whether it happened by the patient falling on the head and shoulders, and thus breaking the arched spine—whether the spines have been crushed against some hard projecting body—or whether the patient has received a blow on the back. By these inquiries, we may be enabled to guess how far the symptoms may be owing to concussion, or to fracture with compression. In the former case, the prognosis is more favourable. If the bone be broken, there is still hope that it may be the spinous processes only which are fractured—" but when it is known that the body or arch of the vertebra is broken, then the case is almost desperate." At the same time, Mr. Bell does not advise his pupils, even in this last event, to follow a line of practice which is to extinguish all hope. " The immediate causes of death, Mr. B. observes, from such a condition of the spine are, first, inflammation of the membranes; secondly, contusion or rupture of the spinal marrow." The great danger, he remarks, in injuries of the spine, is from inflammation.

" Instead of finding a man with fractured spine, lying with his lower extremities motionless and insensible, we may chance to see him jump suddenly out of bed, or struggling under the hands of assistants, or, under the influence of delirium and priapism, talking gaily to the nurse. These are melancholy symptoms, boding the very worst; worse than paralysis—he will die before the fifth day." 8.

Mr. Bell illustrates these remarks by a passage or extract from his case-book.

" '*Sept.* 15*th.* This young man's condition is very threatening, his pulse is 136. He was delirious during the night, and threw himself out of bed. He is now in a state of extraordinary excitement, and although he has full motion of his limbs, yet the spine is undoubtedly broken or crushed, and he will, I fear, die with the symptoms of the last case, and from the same cause, suppuration within the tube of the spine.—*Evening.* He is delirious, and is like a man who is good tempered in his cups: he talks continually, and invites the nurse to bed to him with very gay discourse. His stools and urine still pass involuntarily; pulse 130, weak.—17*th Sept.* It has been necessary to tie him down in bed. He now appears dying: his breathing is very quick and laboured: his pulse hurried, his countenance is sunk, and his tongue is covered with a brown fur.'

"In this case, both the body and ring of the eleventh dorsal vertebra, were fractured." 9.

An instance of *diastasis*, or separation of the vertebræ by violence, with its effects—inflammation of the spinal marrow, is also introduced by our author, in the form of an extract from his note-book.

"'For nearly a week the patient lay without symptoms of injury to the spinal marrow. He could throw his arms and legs about, and retained the urine and fæces. On the eighth day he was seized with convulsions over his whole body. He was relieved by bleeding, and continued sensible, but his jaw was locked. The convulsions returning with violence, he was again bled. A few minutes after this bleeding, his lower jaw moved with great rapidity, and continued moving in an extraordinary manner for nearly five minutes, when all at once he exclaimed he could speak. From this moment he appeared maniacal. His exertions proved that he was not paralytic, it required men to hold him; and he almost sprung out of bed. He passed fæces and flatus with singular force. In the course of an hour he was perfectly composed, and from the time of his first attack of convulsions to his being again sensible, a period of twelve hours elapsed. On the third day he complained of difficulty of using his arm, and two days after he had total palsy of the lower extremities. He lived for a week, retaining much of the character of typhus fever; and the day before his death he recovered sensation in his legs. Dissection showed that the lowest cervical and upper dorsal vertebræ had been separated by violence: the intervertebral substance was destroyed, and pus was not only around the injured part of the spine, but had dropped down through the whole length of the spinal sheath.'" 10.

Mr. Bell next adverts to *subluxation* of the lumbar vertebræ —an accident which, he maintains, so nearly resembles fracture of the spine as to have deceived one of the first surgeons of the age. The accident, he says, happens to young people, and from the operation of a force which, in advanced years, would fracture the bodies of the vertebræ.

"A weight on the head and shoulders overpowering them, and bending them double, the articulating processes of the upper lumbar vertebræ are burst from their connexions; if they again fall into their places, the case is diastasis; but sometimes their edges meet, then it is *subluxation*, they are not restored to their natural position. The body is bent forward, as if the spine were distorted by disease, and the spinous processes of the vertebræ are felt to project, being at the same time deranged from the right line, and leaving an unusual space between them." 11.

The case related by Sir Astley Cooper, at page 551, is considered by Mr. Bell (if we understand him right) to be one of this kind. Sir Astley states, that three or four of the spinous processes had been broken off (the boy fell double under a wheel which he was carrying) and the muscles torn on one side. Mr. Bell questions the *fact* of broken spinous processes, as asserted by Sir Astley—and, therefore, all reasoning or argument is at an end. A man's *inferences* may be questioned—but when the truth of his *statements* is doubted, the Gordian knot is cut, not untied.*

Mr. Bell does not bring forward any case, from his own practice, illustrative of the subject, but he says he has seen one. He begs his pupils to recollect that, in all cases of violence done to the spine, and especially when the vertebræ are thus violently separated in subluxation or diastasis, the theca and membranes may be so injured, that chronic inflammation may be set up, and paralysis of a formidable nature ultimately established. Another cause of death is extravasation of blood.

Our author next comes to fracture of the tube of the spine, and its effects on the spinal marrow. When the injury has been such as to deprive the nerves of their connexion with the brain, the parts below the injury are necessarily paralyzed. Such privation of sense and motion, however, are not the causes of death. In the first place, the bladder becomes distended, and, if not relieved, this distention alone will destroy life by inflammation. The next fertile source of mischief is distention of the intestinal canal, connected with which is the

* Lest we should mistake the meaning of Mr. Bell's remark, or the statement of Sir Astley Cooper, we shall quote the passage in this note.

"A boy was admitted into Guy's Hospital, who had been endeavouring to support a heavy wheel, by putting his head between the spokes, and receiving its weight upon his shoulders; the wheel overbalanced him, and he fell bent double. When he was brought into Guy's Hospital, although he had been perfectly straight before, he had the appearance of one who had long suffered from distorted spine, yet the injury had not produced paralysis of the lower extremities. *Three or four of the spinous processes had been broken off, and the muscles torn on one side,* so as to give an obliquity to the situation of the fractured portions. This boy quickly recovered without any particular attention, and was discharged with the free use of his body and limbs, but he still remained deformed."—*Sir Astley Cooper's Treatise on Dislocations and Fractures,* p 551. On which Mr. Bell remarks:—

"It may be observed that the force was applied to the boy's shoulders, and that he was bent down, and could not recover the upright position. *How, then, could the processes of the vertebræ be broken,* and what is there in the fracture of these processes, which should prevent the lad rising to the erect posture, or cause a permanent distortion of the spine." 11.

That the wheel must have come against the boy's back, is evident from the *muscles being torn off.* In this respect, Sir Astley Cooper could hardly be mistaken, whatever comes of the fractured spinous processes. But surely a foreign body that could tear off the muscles, *might* fracture the projecting spines.

remarkable flaccidity of the abdominal muscles—and the derangement of the respiration in consequence of this defect of action in the abdominal muscles.

The distention of the intestines is accounted for, says Mr. Bell, by the removal of muscular pressure and consequent extrication of gas, in an unnatural quantity from the contents of the intestinal canal—forming what the older authors (and he might have added, the continental authors of the present day,) called meteorismus. We crave permission to observe that, Mr. Bell's explanation is, to us, very far from satisfactory. We think the extrication of gas or flatus in the intestines is much more likely to depend on diminished energy in the nerves of those parts, than on the mere removal of pressure. Who does not daily witness these distressing distentions, in deranged digestion, where relief is sought and obtained—not by increasing, but diminishing the pressure of the abdominal muscles? Who is it that has suffered from dyspepsia, and has not every day been obliged to unloose his waistcoat, and thus procure relief from the distention of stomach and intestines? By these observations we do not mean to deny that relaxation of the abdominal muscles is attended with serious consequences, as is seen in women after parturition—in the operation of paracentesis, &c. That air may be *secreted* too, we have little doubt, else, why should we find it in the pericardium, in the general cavity of the abdomen, and even in the brain. We do not exactly know whether Mr. Bell means to deny or not, this morbid process, by the following phrase—"secretion of air, indeed, is a term which sounds like medical philosophy, &c." p. 15.

Mr. Bell next directs the severity of his strictures against the proposed, and, indeed, performed operation of trephining the spine in certain cases of fracture with depression. We quite agree with our ingenious and talented author, in reprobating the rage among *students* for terrible operations. But surely it is to hospital surgeons that we must look for extending the bounds of surgery, and for performing operations hitherto deemed impracticable. It is not very long since ligature of the subclavian, or even the carotid artery, was looked upon as perfectly chimerical; yet both are now almost annually performed with success. It is therefore dangerous to condemn an operation by *á priori* reasoning, or even by the *event* in one or two cases.

Passing over the offence taken at Sir Astley Cooper's language, as reported in the Lancet, and Mr. Bell's retort courteous, in the volume before us, we come to the question itself, divested of personal feelings, which have too often distorted the judgment, both in medical and surgical investigations.

Mr. Bell objects to the analogy which has been drawn between the operations of trephining the skull and the spine. In the skull, says he, we trephine, because we cannot raise the bone without the application of the instrument.

"But if the ring of the vertebræ be broken down, where is the necessity for applying the trephine? You have the projecting spinous process; you have the inferior and superior edge of the bone on which to place your elevator, or to lay hold of with your forceps. I cannot, for the life of me, imagine any reason why that ring of bone should be trephined, and in two places." 20.

Mr. Bell's objections to the operation in question are still farther developed and illustrated in the following passage.

"A man has received an injury on the spine; his lower extremities are motionless; you suspect that the bone is broken. Aware, in these circumstances, of the danger of bruising or pricking the spinal marrow, you only gently incline the body, and feel the part. It is painful and tumid, and beneath the swollen integuments you think you can feel the spinous process crushed. You know well that you ought not to press and move the part as you might a broken radius. What then, let me ask, are your reflections? 1. That this may be concussion only. 2. That the spinous process may be broken. 3. That extravasation may be, in part, the cause of the symptoms. 4. Anxious to inquire out the nature of the injury, if it appears that the column has been bent or twisted, you fear that it may be a fracture of the body of the vertebræ. Such are the questions you put to yourself; such the difficulties of the diagnosis. You cannot determine whether the man suffers from concussion, from extravasation, from fracture of the tube or of the body of the vertebræ; or whether the spinal marrow be compressed, or torn, or crushed wholly, or divided!" 21.

Now the question is, observes Mr. Bell, whether, in such circumstances, the violence of an operation is to be added to the already existing injury? Our author answers, of course, in the negative. But there are others who think differently. The late Mr. Cline, Jun. proposed and performed the operation. So did Mr. Tyrrell, as our readers know. Sir Astley Cooper, and some very eminent physicians on the continent, have *sanctioned* the operation, under particular circumstances. While hard words and angry feelings, however, pervade the discussions on this point of surgical practice, we can hardly expect to see sound judgment prevail. We shall therefore drop the subject.*

---

* See a dispassionate train of remarks, on this topic, from the pen of Mr. Bedingfield, in the London Medical Repository, for March, 1825.

Three plates are introduced by Mr. Bell, illustrative of fractures of the spine, and executed in his usual masterly style.

2. *Lecture on the Thigh Bone.* Mr. Bell properly insists on the importance of anatomical knowledge to the surgeon, especially when fractures and dislocations are to be treated. Indeed, there is no way in which a surgeon is so commonly disgraced, and ruined in reputation, as by mistakes or ignorance in managing dislocations and fractures of the limbs, and particularly of the thigh.

"When you have left a dislocation unreduced, or have mistaken a fracture for a dislocation, and when, in consequence, your patient is irrecoverably lame, you will find that person beset you like an evil genius; he is ever hanging idle about the market-place, or conspicuous in his gait as he makes his progress along the street: you have committed perhaps only one error, but it seems to be multiplied by his ubiquity, and the same story and the same discussion are every day excited by pity for that man's condition. Nothing but the diligent study of the anatomy will save you from the commission of these faults: forty years' experience in an hospital, and extensive private practice, will not furnish you with such secure rules, as the reconsideration of the place and relation of the thigh-bone, the direction of the forces which act upon it, and the motions which it has to perform." 36.

Mr. Bell first draws the attention of the student to the difference of structure in the extremities and middle of the thigh bone—a difference which greatly modifies the effects of gun-shot wounds of that bone. Thus, a bullet will sink into and lodge in the cancelli of the *extremity*, while it will shiver the centre of the bone into innumerable splinters, and drive them into the surrounding soft parts. These things are to be borne in mind when we meet with a gun-shot wound of the thigh.

"Another instance, proving the importance of distinguishing the structure of the centre and of the extreme parts of the thigh bone, I must press upon your attention. I show you these preparations of the disease called necrosis. You see the shaft of the bone is dead, and a new bone is formed around it. But we have no example of this singular change being wrought upon the extremities of the bone, unless in very peculiar circumstances, while this, you see, is a common case. The cancellated structure of the extremity does not so easily admit of this form of the disease as the diaphysis does. It is the solidity of the diaphysis, with the distinct cavity within, which makes the thigh bone subject to necrosis at its centre. I present you with three specimens of the necrosis of the thigh bone consequent upon amputation (see plate V.) The front of the stump falling into bad suppuration, the matter wrought its way into the meditulium of the bone, and (as in our experiments when the meditulium is destroyed) necrosis followed.

Even in such a case then, if you found a bad stump and a projecting bone, and were to suppose that the whole bone was carious, and to conclude, that to amputate safely, you must take it off at the joint, you would commit one of those errors, for which it is a poor excuse to say, that you knew no better. If you were then forced to divulge your feelings you must express them thus: 'All these various facts and accumulated instances afforded me by my teacher, and the principles drawn from the structure, I have quite neglected.' If you have to amputate, it is only to take off the diseased stump, cut across the bone, and withdraw the sequestra. But see what you may obtain by delay: the portion I now put into your hands was brought away from the face of a stump; you see it is the shaft of the bone wasted and carious, a true sequestra." 38.

For many years past Mr. Bell has been in the habit of pointing out the advantages of free incisions in cases of gunshot wounds. If these be not made, he observes, to give discharge to the matter which collects in the centre of the limb, the matter lodges in the meditullium of the bone, and produces necrosis.

Mr Bell next sets his face against amputation at the hip-joint, at least in the way in which it is commonly performed. He considers it possible (and we believe he is right) to pick out the head of the bone, if that be necessary, after amputating in the common way, as high up as convenient. He does not, however, mean to say that there cannot arise a case for amputation at the hip-joint—" but it is not fracture of the bone by musket shot, nor necrosis of the bone, beginning in the centre of the diaphysis, that forms the case for hip-joint operation."

The subject of *diastasis* is next touched upon by our author, in the following manner.

" A child carried in arms, and throwing itself back in passion, whilst the maid holds it by the thighs, swings the whole weight of the body on the neck of the femur, and sometimes the head parts at the cartilage. There is lameness, and pain, and inflammation; but the case is difficult to be understood, for there is not the crepitus attending fracture, nor the distortion which characterises dislocation. I have not met with an instance of this kind, but cases are on record. I hold in my hand, however, a specimen of diastasis of the lower head of the thigh-bone (see fig. 2, plate IV.) The lad, about thirteen years of age, had fallen through an open floor, and between the joists, so as to twist the leg, and the effect was supposed to be a fracture near the knee-joint. Suppuration took place in the joint, and by the delirious restlessness of the patient, and ulceration of the integuments, the bone was thrust through. A consultation was necessary, and on examination I found it was not a fracture, but that the spongy extremity of the diaphysis projected, whilst he epiphysis retained its connexion with the joint; the lad died. That such a case admits of reunion and cure, you may see by this other specimen, where the lower epiphysis is broken off, and reunited irregularly to the extremity of the shaft (fig. 3, plate IV.). This lad, when getting on the back of a gentleman's carriage, got his leg entangled in the spokes of the wheel, and the force was such as to twist off the epiphysis from the extremity of the diaphysis. His leg was amputated many years afterwards on account of an aneurism, occasioned by the projecting extremity of the shaft." 42.

Mr. Bell places before his pupils eighteen specimens of thigh-bones fractured at the central portion. Of these, sixteen are distorted from the natural form—and all in the same direction—that is to say, the superior portion is drawn forward and upward, and the inferior relatively depressed. "This should remind you," says Mr. Bell, "that there is, in one sense, no accident in this distortion—that the powers acting on the limb are, in their operation, as certain as the direction and insertion of the muscles are uniform." The distortion therefore, is a necessary result of the form of the bone and the operation of the muscles. In amputation, or in fracture of the thigh-bone near the middle, the upper portion of the bone is immediately erected or drawn forward, and the surgeon's attention must be engaged, not to depress the bone, for that produces excitement and spasm, but to counteract its influence in another manner. Our author considers that he has proved the necessity of raising the thigh during the sawing of the bone in amputation; and shewn that, if this be not done, the bone will rise the moment that it is divided, and project from the muscles. An illustrative sketch is introduced, and followed by a quotation from the author's "illustrations of the great operations of surgery."

"*Question.—What is the proper position of the limb during the sawing of the bone?*"

"The pupil, sitting low before the table, holds down the thigh in a horizontal position. But should this be continued during the sawing of the bone? I apprehend not. When the muscles are divided and retracted, and the bone sawn through, all appears well, for the bone is buried in the muscular substance; but when the limb is separated, and the stump is raised, which immediately takes place by an effort of the limb scarcely to be controlled, then the bone projects! Look to the position of the patient when he is laid in bed; does the stump lie in the position of the thigh before operation? No: it is with difficulty kept down; and if not restrained, would be directed upwards, making an angle with the body. In fact, it does stick up, and in proportion as it does so, the bone projects." 44.

Mr. Bell observes that this improvement in the mode of operating, is more followed in the country than in this metropolis—where, when an improvement is made, the surgeons "do not say that it is ingenious or curious, or useful;—but *that is mine.*" Mr. Bell, after making some cursory observations on the double inclined plane, and the proper way to place a fractured limb, goes on to dislocations and fractures of the upper part of the thigh bone. Here Mr. Bell is anxious to prove that many, if not all the doctrines and opinions of Sir Astley Cooper, respecting fracture and non-union of the neck of the thigh bone, were anticipated by, if not stolen from, the writings of his brother John Bell. Men naturally feel for the discoveries of their brothers, their friends, and themselves—but, as we said before, the public care little about the discoverer, provided the discovery be of importance. As chroniclers, however, of the passing events of the day, it would not be proper to pass over the disputed point in question, without some notice. Both parties are our personal friends—but to neither do we owe any obligation beyond that arising out of the amenity of professional intercourse. We shall put it out of the power of either party to accuse us of partiality.

There can be no doubt then that, in Mr. John Bell's publication of the year 1801, the principal points insisted on by Sir Astley Cooper in his late work, respecting fracture of the cervix femoris, have been anticipated, (as far as bibliography is concerned) by that ingenious and unfortunate surgeon. We shall lay Mr. John Bell's claims before our readers as set forth by his brother, in the work before us.

"We now come to the consideration of the neck of the bone: and I shall, in the first place, give you a sketch of what has been done on this subject by my brother, Mr. John Bell. Here let me observe, that his four large volumes of the Principles of Surgery are not suited for your reading until you are well initiated in your profession; and even then they require a commentator: But, on the other hand, it will be a book valuable in succeeding ages, and to all surgeons who are desirous of seeking the principles of their profession. Not only the ingenuity is to be admired which he displays upon this very subject, but also his felicity in illustrating the principles. Among other things, he explains how it happens, that the neck of the thigh bone is abruptly broken off within the capsule; or again, that the head of the bone is extensively fractured, and the portions thrust among the torn membranes and muscles: And he explains at great length, and with every possible variety of illustration, how it happens that the one is curable and the other incurable*. He proceeds thus, page 550: 'When a

"* See p. 549. "Principles of Surgery," 4to. 1801, under the title of "Why is this fracture incurable?"

bone is broken, the soft parts are thickened around it, there is a general soft swelling of the limb, accompanied with a particular tumour surrounding the part, which tumour is hard and firm, and feels as if there were formed round the bone a gland-like mass for the purpose of generating callus. When we break the leg of an animal and examine this thickening, we find the muscles, the cellular substance, and the periosteum thickened, and firmly adhering to the ends of the broken bone: the part is very vascular, and it would appear that this turgescence, swelling, and high action of the vessels, were determined to the generation of bone, which being generated, the action subsides, and the swelling and thickness dissolve.' He continues at page 551: 'But when the rotula or knee-pan is broken, we have to do with a bone which is in very different circumstances.' And at page 553: 'The neck of the thigh bone, which is completely insulated in its natural condition, can form, when broken, none of those conditions with the surrounding parts which should help to make up a mass capable of retaining the bones in close contact, and of assisting in the generation of callus. This is a reason why all our ingenuity is exhausted in vain, why each successive generation has condemned the inventions of the preceding age. All our hopes of succeeding in the cure of fracture in the neck of the thigh bone have been successively abandoned, and we are almost persuaded to subscribe to the bold, unlimited affirmation of Platner, 'Nunquam os eâ parte glutinari posse nec membram in antiquum statum reverti.' The mechanism I have explained is, I fear, a true answer to the question of the celebrated Dessault: 'Why should not the neck heal as well as any other part of this bone?' But why is the neck of the thigh bone, when it does re-unite, surrounded with so clumsy a mass of callus? This also must be explained: for it is a fact, and an interesting one, and must have a place in the account which I am presently to give of the various conditions in which the fractured thigh bone is found after death." 53.

The work from which these excerpts are taken, was dedicated in the year 1801, to Sir Astley Cooper, who is charged by Mr. Charles Bell with very culpable silence as to the work so dedicated. But it is now time to listen to the other side of the question.

In the Preface to the Fourth Edition of his work, Sir Astley Cooper observes as follows:

"I have been accused of publishing doctrines respecting fractures of the neck of the thigh-bone, which differ from those of my medical brethren, and this I am ready and proud to acknowledge; on the other hand, I have heard that I am abused for not having acknowledged that others had previously given similar opinions. To this animadversion I have only to reply, that I began to deliver lectures in the year 1792, and that I never failed in them to give publicity to the opinions which I have here advanced. I have procured early copies of my lectures, taken by some of my students, and I could obtain a great number of others, which shew that my opinions of non-union were those which this book contains."

Sir Astley then introduces extracts from notes taken upwards of 30 years ago, by Mr. Fiske, of Saffron Walden—Mr. Lukin, of Feversham—Mr. Piddock, of Watford—Mr. Pulley, of Bedford—Mr. Weekes, of Hurtspur Point—Mr. Overend, of Sheffield—Mr. Alexander, of Newbury, cum multis aliis, all proving the identity of the opinions then delivered, *vivâ voce,* and now published in the work alluded to. It is quite unnecessary after this, to say that the charge of plagiarism entirely falls to the ground. The charge which Mr. Bell makes against Sir Astley Cooper, of "omitting all mention of his respectable cotemporaries," and of not supporting his reasonings "by the authority of those who have preceded him," is quite another consideration. There are two modes in which an author may come before the public. One is, with the results of his own observation and experience, and without professing to give any thing else. The other, and the more common way is, to bring forward a man's own observations, backed and surrounded by all that has been said on the same subjects, from the days of Hippocrates down to his own times. Of all the evils that ever befell medical literature, this has been the worst. It has multiplied and magnified books to such an extent, and produced such a mass of repetition, as to be the main cause of that very evil of which Mr. Bell complains—*the neglect of reading.* To get at a single new fact (which new fact often turns out to be no fact at all) we suffer the penalty of wading through all that has ever been said, thought, or done on the same topic, for two thousand years. Now we do maintain, that if men of talent and experience, in all ages, had merely given the results of their own observations uncomplicated with those of others, medical science would have been infinitely a gainer, and the individual works of our forefathers would have been a thousand times oftener perused than they now are.* We ask our professional brethren which they would prefer—the simple results of Sir Astley Cooper's or Mr. Bell's experience—or a tissue of quotations from Celsus down to Samuel Cooper?—The answer

* By this observation, we do not mean to undervalue the importance of good *compilations.* These, from time to time, are indispensible. But it by no means follows that profit results from every author who comes forward in the character of originality, turning out a mere compiler from the works of his predecessors. These writers overwhelm the public with books, and bewilder the professed compiler when he attempts to trace property to its original owner.

may be readily anticipated. We think then, that a man of talent and great experience, such for instance, as Sir A. Cooper, or Mr. C. Bell, is to be praised rather than blamed for giving to the public the unadulterated products of his own actual observation, even if these products be unaccompanied by a single name recorded in ancient or modern medicine. Such are our sentiments—and if they be not reasonable, we shall always be ready to listen to reason, and to correct our erroneous impressions.

In respect to the *fact* of non-union of the bone when broken within the capsular ligament, Sir Astley Cooper and Mr. Bell are agreed. As to the *cause* of this non-union, there is discrepancy of opinion, as we might expect. It is well known that Sir Astley Cooper attributes the failure of union to want of apposition, and want of ossific power in the detached portion. Mr. Bell's explanation is as follows: "if the bone be broken within the capsule, it is attended with an increase of colourless effusion into the joint, and the bones remain loose and subject to motion. But if the bone be broken external to the joint, the cellular connexions are torn, and there is bloody effusion; there follows this—inflammation and consolidation of the surrounding parts; the bones are sustained by this mass of inflamed matter; and, in due time, bone is formed in it, and that bone constitutes the medium of re-union." Both explanations are imaginary, or at least matters of mere opinion—and when examined minutely, differ more in words than in things. The reader will take his choice.

"That I may not omit the rule of practice, allow me to state, that when you are by the bed-side of the patient who has fractured the neck of the thigh bone, you cannot decide with absolute cartainty whether the bone be broken in such a manner as to unite or not. You are, therefore, to lay aside these questions, which are for the time so strenuously advocated, of fracture within or without the joint, and to proceed upon the supposition, that the bones may admit of reunion. The limb is to be laid on the double inclined plane, and a belt and compress is to be put round the pelvis, so as at once to offer some resistance to the rising of the trochanter, and press the broken surfaces together. But if it appear that after six weeks there is no reunion, nor such stiffness and swelling as forebode it, we must let the patient rise and use a crutch, lest the parts fall out of use and waste." 61.

Mr. Bell next draws the attention of his pupils to the effect of inflammation of the hip-joint on the position of the thigh bone. Whenever, says he, there is any, the slightest degree of inflammation of the hip-joint, whether it proceed from an injury, or be spontaneous and constitutional inflammation, there is an inclination of the pelvis on the head of the thigh-bone, and the inclination of the trunk from the line of the thigh-bone, increases in proportion to the degree of inflammation. At last, the disease continuing, the affected leg and the trunk will form an angle of 45°, and the head of the femur will be thus raised upon the lip of the acetabulum, and prepared to start out of the socket altogether. This is consecutive dislocation—a subject which Mr. Bell thinks, has not been explained or even understood by any preceding author—his brother not excepted.

"You know very well, gentlemen, that a ligament, to be firm, and white, and strong, must have only its natural degree of vascularity; but if it be inflamed, with its increase of vascularity, it becomes of a gray colour, and softer, and loses its power of resistance. This condition of the ligaments of the hip-joint permits dislocation, but does not cause it. It is the inclination of the body and the leg which throws out the head of the bone from the socket; and, owing to the softening and yielding of the ligaments, there is no check or limit to the distortion, and thus dislocation is *consequent* upon injury." 64.

It is of the highest degree of importance to notice this inclination, from its first to its last degree. In examining a limb which is supposed to be fractured or dislocated, Mr. Bell observes, we are to request the patient to lie on his back, and then putting the heels together, we find whether one leg is shorter than the other—looking carefully, at the same time, to the position of the trunk. By examining the comparative elevations of the ilia, it will be found that there is no actual shor tening of the limb, in many cases, as proved by the obliquity of a line drawn across the pelvis from ilium to ilium.

"There is a sort of puzzle between the shortened and the lengthened limb, while, in reality, the limb is neither shorter nor longer. In Mr. Brodie's work, on diseases of the joints, the lengthening of the limb by disease of the hip is very ingeniously accounted for. To balance the body, without the necessity of resting on the lame hip, the patient projects the foot forward, and across the other foot. This enables him to preserve the body in equipoise on the sound hip-joint. The consequence of this position is, that the pelvis on the diseased side dips lower than on the sound side. To this position of the pelvis the spine is, in time, accommodated; and, when the patient is laid on his back, the lame leg is longest, that is, it projects further, in consequence of the curve of the spine, and inequality of the pelvis." 64.

Mr. Bell assures his pupils that, when they see an inclination of the body upon the head of the thigh-bone, they have an inflammation deep in the hip-joint to encounter—and that when the patient, with an injury in the knee-joint, has a tense corded feeling in the ham-string tendons, a formidable inflammation within the joint has commenced, which must be combated by active measures, otherwise the apparatus of the joint will be destroyed. "If, on the contrary, you see a young woman with swelling and pain of the knee-joint, and do not find this position of the limb and tension of the hamstring tendons, then is the inflammation exterior to the joint, and the case is, probably, only the 'housemaid's knee.'"

Into Mr. Bell's reasonings respecting the shortening and lengthening of a limb, in fracture of the cervix, as illustrated by the near and off wheels of Sir Astley Cooper's carriage, we shall not enter, not being fortunate enough to comprehend them.

Speaking of the knee-joint, Mr. Bell observes that there is one accident, infinitely more important, in comparison, than dislocation, from its greater frequency, and which has been passed unnoticed by Sir Astley Cooper, in his work on dislocations.

"From the same cause, the obliquity of the thigh bone, there is another accident, very frequently taking place, in old women especially; and that is an injury of the internal lateral ligament, consequent upon a false step, by which the body is suddenly jolted upon the thigh bone. For, you see, the thigh bone inclining outwards, the inner condyle is apt to rise from the tibia, and, in consequence, to rupture the internal lateral ligament. I say nothing of that, because it is an obvious accident: but when the injury takes place in a lesser degree, so that the internal ligament of the knee-joint is stretched, sprained, and not lacerated, consequences arise which you can not understand unless your attention has been called to the effects which result from the obliquity of the thigh bone. This ligament, being injured, inflames, softens, and stretches: It allows the knee-joint to sink inwards: the obliquity of the thigh bone is thereby increased, and, of course, all the consequences resulting from that obliquity: every step now gives pain, and the ligament is thus continually injured, and, being kept in a state of inflammation, softens and relaxes, and a permanent lameness and feebleness of the knee-joint is established.

"When you listen to the old lady's story, you hear twenty fancies about it: but having made out, from much talk, that it is placing her foot on the step of the carriage in alighting, or in coming down stairs, that she suffers, you may say, 'Now, madam, I shall touch the part affected:' and when you press on the insertion of the internal lateral ligament, she acknowledges that you have hit the cause. I leave your ingenuity to contrive the means of supporting the joint on the inside." 70.

Mr. Bell directs the attention of the student to the projecting edges of the trochlea of the femur, whose elevated edges keep the patella in its place during the play of that bone upon the knee-joint. The same ridge of bone, he observes, will obstruct the reduction of the patella when dislocated. "The proper method is to press the patella towards the tibia before the attempt be made to bring it in front of the knee-joint."

From the work now closed, we have endeavoured to extract as much as possible of useful matter, out of the mass of criticism in which it is involved. By this line of conduct, we conceive we have rendered our analysis most advantageous to the public and least injurious to the author. Mr. Bell discharges an occasional shot, en passant, at the REVIEWERS. We invite him, in return, to join that corps. There is not a man in England better qualified to form a first rate modern medical critic than Mr. Charles Bell.

